# Molecular and Survival Differences between Familial and Sporadic Gastric Cancers

**DOI:** 10.1155/2013/396272

**Published:** 2013-03-05

**Authors:** Wen-Liang Fang, Shih-Ching Chang, Yuan-Tzu Lan, Kuo-Hung Huang, Su-Shun Lo, Anna Fen-Yau Li, Chin-Wen Chi, Chew-Wun Wu, Shih-Hwa Chiou

**Affiliations:** ^1^Division of General Surgery, Department of Surgery, Taipei Veterans General Hospital, 201 Section 2, Shih-Pai Road, Taipei 11217, Taiwan; ^2^Institute of Clinical Medicine, School of Medicine, National Yang-Ming University, Taipei, Taiwan; ^3^Division of Colorectal Surgery, Department of Surgery, Taipei Veterans General Hospital, 201 Section 2, Shih-Pai Road, Taipei 11217, Taiwan; ^4^National Yang-Ming University, Taipei, Taiwan; ^5^National Yang-Ming University Hospital, 152 Xin-Min Road, Yilan 26042, Taiwan; ^6^Department of Pathology, Taipei Veterans General Hospital, 201 Section 2, Shih-Pai Road, Taipei 11217, Taiwan; ^7^Department of Medical Research and Education, Taipei Veterans General Hospital, 201 Section 2, Shih-Pai Road, Taipei 11217, Taiwan; ^8^Institute of Pharmacology, National Yang-Ming University, Taipei, Taiwan

## Abstract

Mismatch repair (MMR) and germline E-cadherin (CDH1) mutations are two of the major pathways of carcinogenesis in familial gastric cancer (GC). A total of 260 sporadic and 66 familial GC patients were enrolled and molecular and survival differences were compared. Familial GC patients had earlier onset and were diagnosed at an earlier stage and had both a better 5-year overall survival rate and 3-year disease-free survival rate compared with sporadic GC patients. Only in diffuse type GC, the MSI-H phenotype and abnormal MMR protein expression were significantly higher in familial GC than in sporadic GC. In MSI-H GC, MLH1 promoter methylation was slightly higher in sporadic GC than familial GC (50% versus 23.1%), while the frequency of MMR gene mutation was slightly higher in familial GC than in sporadic GC (15.4% versus 3.1%). All of the patients with MMR gene mutation had diffuse type GC. Among familial GC patients with CDH1 mutation, most patients (72.3%) had diffuse type GC. In summary, for familial GC patients, we recommend screening of MSI status and CDH1 mutation especially for diffuse type GC. Because of the low incidence, mutation analysis of MMR gene might be considered in MSI-H familial GC with diffuse type only.

## 1. Introduction

Despite the decreasing incidence worldwide, gastric cancer (GC) is still one of the leading causes of cancer deaths [1]. Two major genomic instability pathways were involved in the pathogenesis of GC: (i) the chromosomal instability (CIN) pathway, which is characterized by gross copy number changes and alterations in chromosomal regions, occurs in at least 60% of cases [2], and (ii) the microsatellite instability (MSI) pathway, which is characterized by alterations in the length of repetitive microsatellite sequences, accounts for 10%–20% of cases [3–5].

In the MSI pathway of gastric carcinogenesis, mutations of hMLH1 were reported in approximately 0%–7.3% of MSI-H GC [6, 7]. However, hMLH1 silencing due to promoter methylation has been reported to be associated with the development of more than 50% of MSI-H GC [8–12].

The incidence of hereditary diffuse gastric cancer (HDGC) in the general population has not yet been clearly defined, but HDGC most likely accounts for only 1%–3% of GCs. Direct evidence shows that hereditary GC with germline E-cadherin (CDH1) mutations is an autosomal dominant inheritance [13]. However, CDH1 mutations account for only 1%–3% of all GCs. Only one-third to one-half of families with a strong history of diffuse GC are associated with CDH1 mutations. The definition of HDGC in 1999 by the International GC Linkage Consortium (IGCLC) was as follows: (i) in the first- or second-degree relatives, two or more cases of DGC diagnosed before 50 years old or (ii) at any age, three or more cases diagnosed as DGC [14, 15]. Nearly 30%–46% of the patients fulfilling the previous criteria carry the CDH1 mutations. The IGCLC criteria have been modified, and patients who were diagnosed with signet-ring carcinoma of the colon or lobular breast cancer were included [16]. However, only 11% of the patients fulfilling the modified criteria carry the CDH1 mutations.

A family history was reported in only 10% of GC cases [17]. Familial GC was reported to be associated with a worse prognosis than sporadic GC [8, 18]. The definition of familial GC includes familial diffuse GC (the so-called HDGC) as well as familial intestinal GC (FIGC) [14]. In countries with high incidence of GC, such as Japan and Portugal, the diagnostic criteria of FIGC were as follows: (i) at least three relatives have intestinal GC and one of them is a first-degree relative of the other two; (ii) at least two generations have GC; or (iii) in one of the relatives, GC should be diagnosed before 50 years old. In countries with low incidence of FIGC, such as USA and UK, the definition was as follows: (i) at least two first/second-degree relatives have intestinal GC, one diagnosed before 50 years old, or (ii) three or more relatives with intestinal GC at any age.

To our knowledge, only a few studies have investigated the MSI status and MLH1 methylation in GC patients with a family history [5, 7, 19–24]. Leite et al. [7] reported that the MSI status and MLH1 methylation were similar between sporadic and familial GCs; however, no MMR gene mutation could be identified in their studies. In Taiwan, there has been no report regarding genetic mutations (including MMR and CDH1 mutations) in GC patients with a family history. The aim of this study is to compare the clinicopathological characteristics, MSI phenotype, immunohistochemical (IHC) stains of MMR proteins, MLH1 promoter methylation, and genetic mutations between familial and sporadic GCs.

## 2. Materials and Methods

A total of 326 GC patients who received surgery between May 1988 and December 2004 were collected from Taipei Veterans General Hospital and included in this study. The information of family history of GC was obtained from the records of the patients and their families. The study was approved by the Institutional Review Board at the Taipei Veterans General Hospital. The written informed consent was obtained from all patients enrolled. The exclusion criteria include (i) patients with a history of gastric surgery or a pathological diagnosis other than adenocarcinoma and (ii) patients belonging to families of hereditary nonpolyposis colorectal cancer (HNPCC).

Patients enrolled in this study were classified and grouped as having either sporadic GC or familial GC. The definition of familial GC in the present study was (i) two or more cases of GC in the first- or second-degree relatives, including at least one patient of GC diagnosed before 50 years old or (ii) three or more cases of GC in first- or second-degree relatives diagnosed at any age. The definition of sporadic GC was patients without a family history of GC.

The pathological staging of cancer was according to the 7th AJCC/UICC TNM classification [25]. The data were collected prospectively and recorded using a computer. The patients were regularly followed up, and the database was updated regularly.

**Figure 1:**
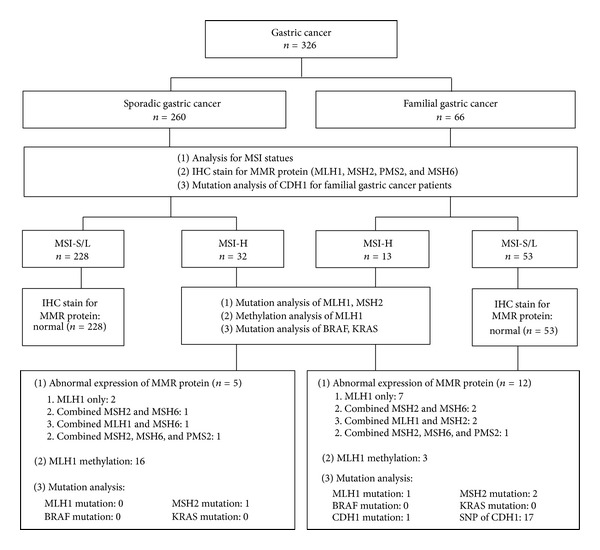
The flowchart of this study.

Microsatellite instability analysis and IHC stains for MMR protein were performed for all the 326 patients enrolled. Patients with MSI-H tumors (32 sporadic GC and 13 familial GC) were analyzed for MLH1 methylation and genetic mutations of MLH1 and MSH2. CDH1 mutations were performed for 66 familial GC patients (Figure 1).

### 2.1. Microsatellite Instability Analysis

The DNA of normal and tumor tissues was extracted from the formalin-fixed, paraffin-embedded (FFPE) tissues or from fresh frozen tissues stored at −80°C or liquid nitrogen. After the DNA was purified by the QIAamp Tissue Kit (QIAGEN GmbH, Germany), the quantitative DNA analysis was performed by measuring the optical density (OD) at wavelengths of 260 nm and 280 nm. The DNA quality was confirmed by the ratio of OD260/280. 

The purified DNA was amplified by using a fluorescent polymerase chain reaction (PCR). Five reference microsatellite markers, including D5S345, D2S123, D17S250, BAT25, and BAT26, were used for the determination of MSI [26]. PCR products were denatured and analyzed by electrophoresis on 5% denatured polyacrylamide gels. The results were analyzed by an ABI 3730 automated sequencer (Applied Biosystems, Foster City, CA, USA). As reported in a previous study [27], the presence of novel alleles observed among the PCR products from tumor DNA that were not seen among the PCR products from the corresponding normal DNA was scored as MSI at that particular locus. Samples with ≥2 loci of instability with 5 markers were defined as MSI-H. Samples with one MSI or without MSI were defined as MSI-L/S. 

### 2.2. Immunohistochemical Stains

IHC stains for MLH1, MSH2, MSH6, and PMS2 were performed for paraffin-embedded tissue. Paraffin-embedded tissue sections (4 mm thick) were stained with antibodies for MLH1 (1 : 10 dilution; Pharmingen, San Diego, CA, USA), MSH2 (1 : 200; Oncogene Research Products, La Jolla, CA, USA), MSH6 (1 : 300; Transduction Laboratories, San Diego, CA, USA), and PMS2 (C20; 1 : 400; Santa Cruz Biotechnology, Santa Cruz, CA, USA). Negative control slides were made without the primary antibody. 

### 2.3. Methylation Analysis of MLH1

Methylation of the hMLH1 promoter was analyzed from GC tumor tissues by using a methylation-specific PCR method. The genomic DNA was modified by sodium bisulfite [28], and the sequences were amplified with different methylated and unmethylated primers [29].

### 2.4. Detection of Mutations for MLH1 and MSH2

Analysis of mutations of MLH1 and MSH2 genes was performed for MSI-H GC. The DNA was extracted from the normal tissue and amplified by PCR and sequenced with primers that have already been applied in the previous studies [30, 31]. For each round of PCR amplification, a negative control template containing no DNA was included. The PCR products were analyzed by the automated sequencer (ABI Prism 3100 Genetic Analyzer). Each sample was sequenced on both of the sense and antisense strands. A second sequencing procedure with new PCR products confirms each mutation.

Nonsense, missense, and frameshift mutations were identified by comparing the obtained sequence with the known sequence. Nonsense and frameshift mutations were considered as pathogenic. Missense mutations in the MMR genes that did not result in abnormal expression of MMR proteins were considered to be polymorphisms [32, 33]. 

### 2.5. Detection of Mutations for BRAF and KRAS

Mutation analysis for BRAF (V599E) and KRAS was performed for MSI-H GC (Figure 1). PCR reactions took place in a volume of 25 *μ*L containing 20 ng genomic DNA template, 0.2 *μ*M of each PCR primer, 0.2 mM dNTPs, PCR buffer, and 1U Taq DNA polymerase. Thirty-five cycles of 30 s at 95°C, 30 s at a primer pair annealing temperature of 55°C, and 60 s at 72°C were performed in programmable thermocyclers (GeneAmp PCR System 2700, ABI). A 3 *μ*L aliquot of each PCR reaction was carried out on a 2% agarose gel. The remaining 17 *μ*L of the PCR product was submitted to purification using a FavorPrep GEL/PCR Purification Mini Kit (FAVORGEN), and the products were eluted in 30 *μ*L Elution Buffer. Sequencing was performed using the BigDye Terminator V3.0 (ABI), data collection mode on an ABI 3730 capillary sequencer.

### 2.6. Detection of Mutations for CDH1

 The DNA was extracted from the normal tissue of the familial GC tissue and further amplified by PCR reactions. The sequences of all the primers used in this study and their annealing temperatures are listed in Supplementary Table 1 available online at http://dx.doi.org/10.1155/2013/396272 PCR reactions was took placed in a volume of 25 *μ*L containing 20 ng of genomic DNA template, 0.2 *μ*M of each PCR primer, 0.2 mM dNTPs, PCR buffer, and 1U Taq DNA polymerase. Thirty-five cycles of 30 s at 94°C, 30 s at a primer pair specific annealing temperature of 50–55°C, and 30 s–90 s at 72°C were performed in programmable thermocyclers (GeneAmp PCR System 2700, ABI). A 3 *μ*L aliquot of each PCR reaction was carried out on a 2% agarose gel, and the size, purity, and quantity of each PCR product were confirmed. The remaining 17 *μ*L of the PCR products was submitted to purification using FavorPrep GEL/PCR Purification Mini Kit (FAVORGEN), and the products were eluted in 30 *μ*L Elution Buffer. Sequencing was performed using the BigDye Terminator V3.0 (ABI), data collection mode on an ABI 3730 capillary sequencer.

### 2.7. Statistical Analysis

The results in the tables are shown as the mean values ± standard deviation. A chi-squared test with Yates' correction was used to analyze categorical variables. Student's *t*-test was used to compare quantitative variables between groups. SPSS version 16.0 (SPSS Inc., Chicago, IL, USA) was used for statistical analysis.

The overall survival was measured from the operation date to the date of death or the final followup. The disease-free survival was defined as the length of time after surgery for gastric cancer during which a patient survives without tumor recurrence. The distributions of overall survival and disease-free survival were estimated using Kaplan-Meier method. The differences between the curves were tested using a two-tailed log-rank test. Cox-proportional hazards models were used to explore the association of clinical parameters with overall survival. A *P* value of <0.05 was considered to be statistically significant.

## 3. Results

### 3.1. Clinicopathological Characteristics

Because the biological behaviors are different between diffuse type and intestinal type in GC, we separate diffuse type and intestinal type for analyzing the difference between sporadic and familial GCs.

As shown in Table 1, for diffuse type GC, familial GC was associated with younger age, less male predominance, smaller tumor size, more well-defined gross appearance, an earlier tumor stage, and a significantly higher frequency of MSI-H tumors as compared to sporadic GC (28% versus 6.5%).

In Table 2, for intestinal type GC, familial GC was associated with younger age, less male predominance, smaller tumor size, more medullary stromal reaction types and an earlier tumor stage as compared to sporadic GC. The frequency of MSI-H tumors was similar between sporadic and familial GCs (16.3% versus 14.6%).

**Figure 2:**
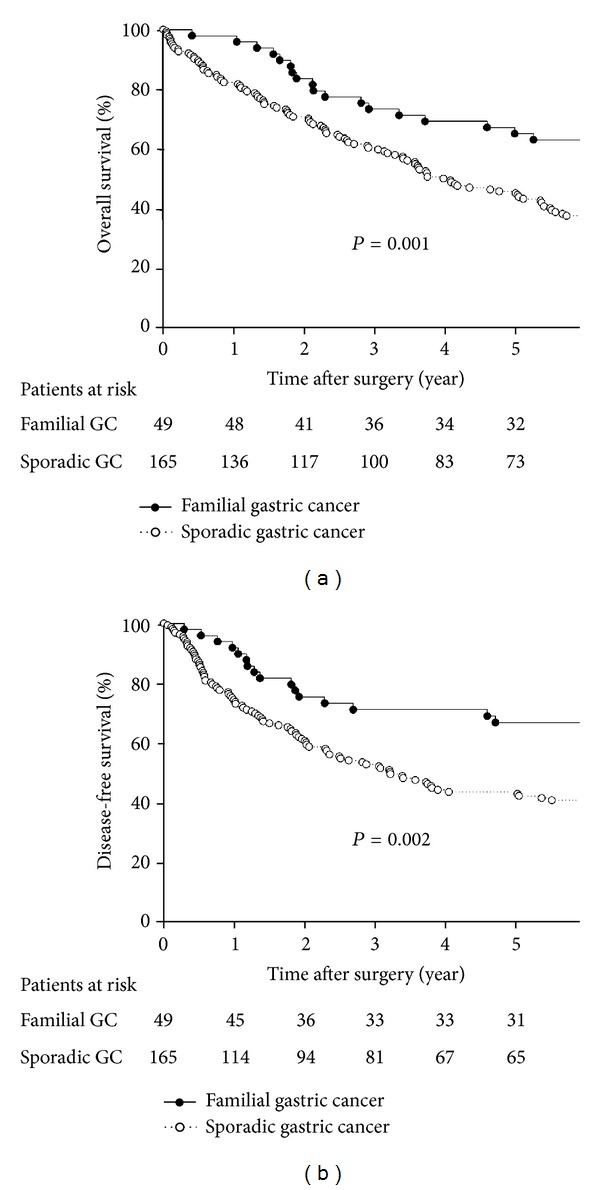
(a) Familial GC was associated with a better 5-year overall survival rate than sporadic GC (65.3% versus 45.4%, *P* = 0.001). (b) Familial GC was associated with a better 3-year disease-free survival rate than sporadic GC (71.1% versus 52.9%, *P* = 0.002).

The overall survival rate was analyzed for GC patients after curative resection. Familial GC patients had a better 5-year overall survival rate than sporadic GC patients (65.3% versus 45.4%, *P* = 0.001, Figure 2(a)). Furthermore, familial GC patients also had a better 3-year disease-free survival rate than sporadic GC patients (71.1% versus 52.9%, *P* = 0.002, Figure 2(b)).

Univariate analysis showed that age, gender, tumor size, lymphovascular invasion, stromal reaction type, family history, MSI status, pathological T category, N category, and TNM stage were associated with survival. Multivariate Cox proportional-hazards model using the forward logistics regression stepwise procedure for the analysis of overall survival showed that gender, pathological TNM stage, and MSI status were independent prognostic factors (Table 3).

### 3.2. Analysis of MSI Status

 Of the total 326 patients, 45 patients (13.8%) had MSI-H GC. MSI-H GC was associated with more tumors located over the distal third of the stomach compared with MSI-L/S GC (68.9% versus 50.2%, *P* = 0.019).

The 5-year overall survival rate of the MSI-H patients was better than that of the MSI-L/S patients after curative surgery (68% versus 47.6%, *P* = 0.032). 

### 3.3. Immunohistochemical Stains for MMR Proteins

 IHC stains for MLH1, MSH2, MLH6, and PMS2 proteins were performed for all the 326 patients enrolled (Figure 1). None of the MSI-L/S tumors had abnormal IHC stains for MMR proteins.

As shown in Figure 1, among the 260 sporadic GC patients, 12 (4.6%) patients had abnormalities on IHC analysis of the MMR protein. Among them, seven patients had abnormal MLH1 stains only; two patients had combined abnormal stains of MLH1 and MSH2; three patients had combined abnormal stains of MSH2, and MSH6.

Among the 66 familial GC patients, six patients (9.1%) had abnormalities on IHC analysis for the MMR protein. Among them, 2 patients had abnormal stains of MLH1 only; one patient had combined abnormal stains of MLH1 and MSH2; one patient had combined abnormal stains of MSH2 and MSH6; one patient had combined MLH1 and MSH6; one patient had combined abnormal stains of MLH1, MSH2, MSH6 and PMS2.

As shown in Table 1, for diffuse type GC patients, abnormal IHC stains for MMR protein were significantly higher in familial GC than in sporadic GC (12% versus 1.9%, *P* = 0.047). In Table 2, for intestinal type GC, the frequency of abnormal IHC stains for MMR protein was similar between familial GC and sporadic GC (7.3% versus 6.5%, *P* = 1.000).

### 3.4. Methylation of the MLH1 Promoter

Methylation of the MLH1 promoter was performed for the 45 MSI-H GC patients, including 32 MSI-H sporadic GC and 13 MSI-H familial GC. Among the 45 MSI-H GC, methylation of the MLH1 promoter was identified in 50% (16/32) of sporadic GC and 23.1% (3/13) of familial GC (*P* = 0.182).

### 3.5. Mutation Analyses for MLH1 and MSH2

Mutation analyses for MLH1 and MSH2 were performed for the 45 MSI-H GC patients. A total of 3 patients were identified as having MMR gene mutations, including one familial GC patient with both MLH1 and MSH2 mutations, one familial GC patient with an MSH2 mutation, and one sporadic GC patient with an MSH2 mutation. All the three patients with MMR gene mutation had diffuse type GC. None of the patients with MLH1 methylation had mutations of MLH1 or MSH2. Among the 45 MSI-H GC patients, the frequency of MMR gene mutations was higher in familial GC (2/13, 15.4%) than sporadic GC (1/32, 3.1%). 

### 3.6. The Correlation between MLH1 Expression, Promoter Methylation, and Mutation

 As shown in Table 4, abnormal expression of IHC stain for MLH1 was observed in 12 patients, including 3 sporadic GC and 9 familial GC. Among the 3 sporadic GC patients, all had MLH1 methylation and none had MLH1 mutation. Among the 9 familial GC patients, 5 patients had MLH1 methylation and one had MLH1 mutation. The only one familial GC patient with MLH1 mutation did not have MLH1 methylation. MLH1 promoter methylation and MLH1 mutation play a different role in the cause of abnormal MLH1 expression.

### 3.7. Mutation Analyses for BRAF and KRAS

 Mutation analyses for BRAF and KRAS were performed for the 28 MSI-H GC patients. However, no BRAF or KRAS mutation was identified.

### 3.8. Analysis of CDH1 Mutations for Familial GC Patients

**Figure 3:**
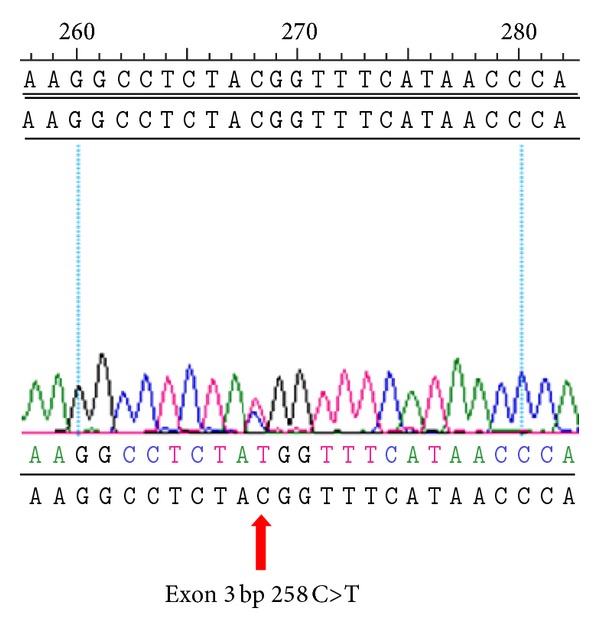
Chromatograms of one patient with pathogenic exon 3 mutation. An arrow indicates the position of the mutation.

Analysis of CDH1 mutations was performed for the 66 familial GC patients. Among them, 18 (27.3%) had CDH1 germline sequence alterations, including 9 patients with rs1801552 (2076T>C, exon 13) alterations, 3 patients with rs33964119 (2253C>T, exon 14) alterations, 1 patient with an exon 3 mutation, and 5 patients with both rs1801552 (exon 13) and rs33964119 (exon 14) alterations. Of the 18 patients with CDH1 germline sequence alterations, only one patient (5.6%) with an exon 3 mutation in codon 90 (268C>T) had an amino acid mutation (Thr to Met, Figure 3); single nucleotide polymorphism was observed in the other 17 patients. Among the 18 patients with CDH1 germline sequence alterations, 13 (72.3%) had diffuse type GC.

### 3.9. The Correlation between CDH1 Mutation and MSI Status in Familial GC

 As shown in Table 5, among the 13 MSI-H familial GC patients, 4 (30.8%) patients had CDH1 mutation, while 14 (26.4%) out of the 53 MSI-L/S familial GC patients had CDH1 mutation. There was no significant difference between the frequency of CDH1 mutation in MSI-H and MSI-L/S familial GC patients (*P* = 0.739).

## 4. Discussion

Our data showed that familial GC patients were diagnosed earlier and associated with a better 5-year overall survival rate (65.3% versus 45.4%, *P* = 0.001) and a better 3-year disease-free survival rate (71.1% versus 49.1%, *P* = 0.002) compared with sporadic GC patients. Only in diffuse type GC, the MSI-H phenotype and abnormal MMR protein expression were significantly higher in familial GC than in sporadic GC. MSI status was one of the independent risk factors affecting survival. For MSI-H GC, MLH1 promoter methylation was slightly higher in sporadic GC than familial GC (50% versus 23.1%). Because this is a retrospective study, selection bias might happen and affect the results. To our knowledge, this study is the first investigating the MSI status, mutation analysis of the MMR gene, and the CDH1 gene in familial GC in Taiwan.

MLH1 promoter methylation was reported to be responsible for more than 50% of MSI-H GC [4, 7, 23]. Leite et al. [7] reported that the frequency of MLH1 promoter methylation is similar between familial and sporadic GCs with MSI-H (71.4% versus 79.3%). Although not statistically significant, our results showed higher frequency of MLH1 promoter methylation in sporadic GC than in familial GC with MSI (50% versus 23.1%). It seems that MLH1 promoter methylation plays a less important role in the cause of MSI-H in our familial GC compared to the sporadic GC. However, it seems that the frequency of MLH1 methylation was lower in our series than the results in the study of Leite et al. [7]. Racial and environmental factors might have an impact on the frequency of MLH1 promoter methylation. Furthermore, we also analyzed the correlation between MLH1 expression, methylation, and mutation (Table 4). We found that the abnormal expression of MLH1 in the three sporadic GC patients was all related to MLH1 methylation. In contrast, the cause of abnormal expression of MLH1 was 55% by MLH1 methylation and 11.1% by MLH1 mutation in familial GC. Consequently, the mechanism of inactivation of MLH1 was mainly epigenetic in both sporadic and familial GCs. A larger sample size may be needed to compare the difference in MLH1 promoter methylation between familial and sporadic GCs.

Our results showed that the frequency of MLH1 or MSH2 gene mutation was slightly higher in familial GC than in sporadic GC (15.4% versus 3.1%) in MSI-H GC. All the three patients with MMR gene mutation had diffuse type GC. In the series of Leite et al. [7], analysis of MMR gene mutation was performed for two patients with simultaneous loss of MLH1 and PMS2 protein in IHC stain; however, no alteration of MLH1 or MSH2 gene was detected. Moreover, Leite et al. [7] also reported no significant difference of MSI status between familial and sporadic GC. In diffuse type GC, the frequency of MSI-H was zero in familial GC and 13.4% in sporadic GC. However, Pedrazzani et al. [5] and Kanemitsu et al. [22] reported significantly higher frequency of MSI-H in familial GC than in sporadic GC. Compared to other series, the novel findings of our results were significantly more MSI-H tumors in familial GC than sporadic GC only in diffuse type GC. Although MSI-H tumors were reported to be more frequent in intestinal type GC, our results showed that MMR gene mutation among the MSI-H tumors mainly occurred in the diffuse type GC. These results are interesting and might be a clue for future investigation of the MMR gene mutation in familial GC. Because the mutation rate of MMR gene is relatively low and the cost for mutation analysis is high, focusing on the diffuse type GC might be more cost-effective than mass screening for all MSI-H GC. According to our results, we recommend screening of MSI status in diffuse type familial GC. Because of the low incidence, analysis of MMR gene mutation might be considered for MSI-H familial GC with diffuse type only. 

Ye et al. [8] matched their sporadic GC patients with their familial GC patients for age and TNM stage, and they concluded that familial GC was associated with a worse prognosis than sporadic GC. However, early onset and early diagnosis of familial cancer were ignored simultaneously, which might also cause selection bias. Our data showed that familial GC patients were diagnosed at an earlier tumor stage and had a younger age, which might be the reason why our familial GC patients had a better prognosis. It seems that GC patients with a family history tend to pay attention to the symptoms and seek medical help earlier than those without a family history, which was also observed in colorectal cancer [34]. Our results showed that family history is associated with a better prognosis only by univariate analysis. The possible reason might be that patients with a family history are usually diagnosed in the early stage, and the importance of family history in the prognosis might be replaced by the TNM stage in the multivariate analysis. Furthermore, as shown in Table 1, about 25.8% of our familial GC patients were diagnosed at stage IV, for whom curative surgery was impossible; consequently, this group of patients had a worse prognosis. We should make effort to strengthen the health education and perform extensive screening for the relatives of GC patients, especially for the first- and second-degree relatives, in order to detect GC earlier for them.

Our results demonstrated that MSI status was an independent risk factor of GC after curative surgery, which was also mentioned in our previous study [35] and similar to the results of some series [36, 37]. However, other authors [19, 38] reported that MSI was not associated with survival. Because the frequency of MSI was as low as 8%–25% in these series and a relative small number of patients with MSI tumor were analyzed, a larger sample size is needed to clarify the role of MSI in survival.

Some authors reported that the MSI status was associated with a family history of GC [5, 19, 22], but others reported the opposite [20, 21, 24]. Our data showed a higher percentage of MSI-H tumors in familial GC than in sporadic GC, which was only observed in diffuse type GC. In diffuse type GC, our data showed that familial GC was associated with a better 5-year overall survival rate than sporadic GC (44% versus 28%, *P* = 0.028). The reason might be due to a higher percentage of MSI-H tumors in our familial GC with diffuse type, and MSI-H tumors were associated with a better prognosis than MSI-L/S tumors. This divergence between each study is most likely due to the different criteria used for the definition of family history and the small size of patients with a family history. Furthermore, some studies included both intestinal and diffuse type GCs in their analysis, which might cause different results. Further meta-analysis of a larger number of patients and separating the diffuse and intestinal type GC for analyzing the association between the family history and MSI status might provide more reliable results.

BRAF and KRAS mutations were reported in only 2% of GC patients and, specifically, only in advanced GC [39]. BRAF mutation in GC could exclude germline mutations of MMR. Screening for BRAF in MSI-H GC could decrease the waste for an expensive mutation analysis. As a result, we also analyzed the BRAF and KRAS mutations in our patients. However, we detected neither BRAF nor KRAS mutations in our patients. BRAF and KRAS mutations may not play an important role in our GC patients.

 Our data showed that the majority (94.4%, 17/18) of CDH1 gene alterations were silent mutations, or the so-called synonymous SNPs, including rs1801552 and rs33964119, which have been reported in some series [9, 10]. A silent, single nucleotide polymorphism (SNP) in a gene is one that creates a codon that is synonymous to the wild-type codon. However, this synonymous codon substitution may lead to different kinetics of mRNA translation, thus yielding a protein with different final structure and function [40]. *In silico* analysis suggests that these sequence alterations may affect splicing and protein conformations [41]. Moreover, we identified one patient with an exon 3 mutation at codon 90 (268C>T), which had never been reported in the literature. Further study of this newly found mutation in the CDH1 gene is required. Our results showed that among our familial GC patients with CDH1 mutation, 72.3% patients had diffuse type GC. As a result, routine screening of CDH1 mutation is recommended in diffuse type familial GC.

 The two major genetic instability pathways of the carcinogenesis of gastric cancer were CIN and MSI. CDH1 mutation was reported to be involved in the pathogenesis in familial GC. In this study, we also analyzed the correlation between MSI status and CDH1 mutation in familial GC. However, there was no difference in the frequency of CDH1 mutation between MSI-H and MSI-L/S GC. It seems that there is no relationship between CDH1 mutation and MSI in familial GC. The result is reasonable because CDH1 mutation and MSI are involved in different pathways for carcinogenesis of GC.

As shown in Figure 1, we identified the MLH1 promoter methylation, MMR gene mutation, and CDH1 mutation in sporadic and familial GCs. However, these mutations can only explain the carcinogenesis of some of our patients, especially in familial GC. There are still unknown genes involved in the pathogenesis of GC, and additional studies are necessary to identify and characterize these genes. As a matter of course, there is still a lot of space for us to explore GC in the future.

## 5. Conclusion

In conclusion, familial GC was associated with an early stage at diagnosis and a better prognosis compared with sporadic GCs. Our results display the molecular and survival differences between sporadic and familial GC. Because of the relatively higher accumulation of GC in patients with a family history, annual upper gastrointestinal endoscopic examinations are recommended in the relatives of familial GC patients. For GC patients with mutations of CDH1 or MMR genes, genetic screening of their relatives is recommended, especially for the first- and second-degree relatives.

## Supplementary Material

Supplementary Table 1: lists the primer sequences used in the study for detection of CDH1 mutations and their annealing temperatures.

## Figures and Tables

**Table 1:** Comparison of clinicopathological characteristics between diffuse type GC cases of sporadic and familial GCs.

	Sporadic GC	Familial GC	
	(*n* = 107)	(*n* = 25)	*P* value
	*n* (%)	*n* (%)	
Age (years)	65.4 ± 12.8	54.1 ± 15.0	<0.001
Gender (M/F)	77/30	12/13	0.032
Tumor maximal size (cm)	7.9 ± 3.1	5.8 ± 2.4	<0.001
Gross appearance			
Well-defined	16 (15)	12 (48)	
Ill-defined	91 (85)	13 (52)	0.001
Lymphovascular invasion			
Absent/present	10/97	11/14	<0.001
Stromal reaction type			
Medullary	18 (16.8)	7 (28)	
Intermediate	36 (33.6)	7 (28)	
Scirrhous	53 (49.5)	11 (44)	0.435
Pathological T category			
T1/T2/T3/T4	4/6/26/71	9/2/1/13	<0.001
Pathological N category			
N0/N1/N2/N3	15/15/17/60	9/2/4/10	0.074
Pathological TNM stage			
Stage I	5 (4.7)	9 (36)	
Stage II	11 (10.3)	2 (8)	
Stage III	46 (43)	7 (28)	
Stage IV	45 (42)	7 (28)	<0.001
MSI status			
MSI-L/S	100 (93.5)	18 (72)	
MSI-H	7 (6.5)	7 (28)	0.005
IHC stain for MMR protein			
Normal	105 (98.1)	22 (88)	
Abnormal	2 (1.9)	3 (12)	0.047

**Table 2:** Comparison of clinicopathological characteristics between intestinal type GC cases of sporadic and familial GCs.

	Sporadic GC	Familial GC	
	(*n* = 153)	(*n* = 41)	*P* value
	*n* (%)	*n* (%)	
Age (years)	70.8 ± 9.8	60.2 ± 13.4	<0.001
Gender (M/F)	124/29	24/17	0.006
Tumor maximal size (cm)	6.3 ± 2.3	4.9 ± 3.3	<0.001
Gross appearance			
Well-defined	64 (41.8)	21 (51.2)	
Ill-defined	89 (58.2)	20 (48.8)	0.293
Lymphovascular invasion			
Absent/present	23/130	19/22	<0.001
Stromal reaction type			
Medullary	16 (10.5)	14 (34.2)	
Intermediate	104 (68)	19 (46.3)	
Scirrhous	33 (21.5)	8 (19.5)	0.001
Pathological T category			
T1/T2/T3/T4	15/22/36/80	16/2/7/16	<0.001
Pathological N category			
N0/N1/N2/N3	39/25/29/60	19/5/3/14	0.047
Pathological TNM stage			
Stage I	24 (15.7)	16 (39)	
Stage II	33 (21.6)	7 (17.1)	
Stage III	46 (30.1)	8 (19.5)	
Stage IV	50 (32.7)	10 (24.4)	0.012
MSI status			
MSI-L/S	128 (83.7)	35 (85.4)	
MSI-H	25 (16.3)	6 (14.6)	1.000
IHC stain for MMR protein			
Normal	143 (93.5)	38 (92.7)	
Abnormal	10 (6.5)	3 (7.3)	1.000

**Table 3:** Univariate analysis and multivariate analysis of factors affecting overall survival of GC patients after curative surgery.

	Univariate analysis		Multivariate analysis	
	OR (CI)	*P* value	OR (CI)	*P* value
Age (<65, ≥65 years)	1.52 (1.04–2.23)	0.031		
Gender (male, female)	0.62 (0.42–0.93)	0.020	0.66 (0.44–0.98)	0.038
Tumor size (<5 cm, ≥5 cm)	2.15 (1.47–3.13)	<0.001		
Lauren's classification (intestinal/diffuse)	0.85 (0.60–1.20)	0.347		
Lymphovascular invasion (−, +)	3.21 (1.99–5.17)	<0.001		
Stromal reaction type (medullary, intermediate, and scirrhous)	1.47 (1.15–1.87)	0.002		
Family history (−, +)	0.43 (0.27–0.71)	0.001		
MSI status (MSI-L/S, MSI-H)	0.52 (0.28–0.96)	0.035	0.50 (0.27–0.93)	0.029
TNM stage (I, II, III)	2.43 (1.89–3.13)	<0.001	2.44 (1.89–3.14)	<0.001

OR: odds ratio, CI: confidence interval.

**Table 4:** The frequency of MLH1 promoter methylation and MLH1 mutation in GC patients with abnormal MLH1 expression.

	Sporadic GC	Familial GC
	(*n* = 3)	(*n* = 9)
	*n* (%)	*n* (%)
MLH1 promoter		
Methylation	3 (100)	5 (55.6)
Unmethylation	0	4 (44.4)
MLH1 mutation		
Yes	0	1 (11.1)
No	3 (100)	8 (88.9)

**Table 5:** The frequency of MSI status and CDH1 mutation in familial GC patients.

	MSI-H	MSI-L/S	
	(*n* = 13)	(*n* = 53)	*P* value
	*n* (%)	*n* (%)	
CDH1 mutation			
Present	4 (30.8)	14 (26.4)	
Absent	9 (69.2)	39 (73.6)	0.739
