# Supplementary material for: Molecular and Survival Differences between Familial and Sporadic Gastric Cancers
Source: Biomed Res Int. 2013 Mar 5;2013:396272. doi: 10.1155/2013/396272 (PMC3603157; doi:10.1155/2013/396272)
Supplement: Supplementary file 1 — Supplementary Table 1: lists the primer sequences used in the study for detection of CDH1 mutations and their annealing temperatures. [file 396272.f1.pdf]

Supplementary table 1. Primer sequences used in the study for detection of CDH1 mutations and their annealing temperatures.

Exon	Forward	Reverse	Tm (˚C)
1, 2	TGTGAGCTTGCGGAAGTCA	GCCAAGGAGGGAGCTTG	55
3	GTGTTTGGTTTTGTGGGAGTCT	GTCAACGGTACCAAGGCTGA	55
4,5	GTCTGGCTAGGTTGGACTGTTAGA	GGATCCAGCATGGGTTGAC	55
6	TCTCAGAGCCTAGGAAGGTGTG	CAAGAAGTTCTGTCCGTAGGAAGG	55
7	AGGGCAGAATTGGATTAAG	GGGCACAAGCTTAGATGCAA	50
8	CGTGCCTAGAAGACAGGCAG	CTTCACAATCTTGCACCAATTG	50
9	TGTCACATCTTCTCCTTGAAGCT	CCACATGGTCCACTACAATCTG	50
10	GAAAGTCATGGCAGAAACCACA	AGGGAACAGGTGAAAGGAGCA	55
11	TGTTGACCAGGCTGGTCTC	ATGCATGTTATTTGGGTGACG	55
12	CACTGAAGAGCCAGGACAAGAT	ACAGAAGGGACAAGGAAGCAAG	50
13	CTGCTCTCTTCACTCGGCTT	TCTATTTTATGGAGGCTGGCA	50
14	AGGCAGCTAGTGGCTGTCTAACT	CTCGCTCACTGAGGTGCAGA	55
15	GCAGTGAAGGCATCATCCAAC	GCTCAGGCAAGCTGAAAACA	50
16	CACAAGTCTGGGTGCATTGTC	ATCTCAAGGGAAGGGAGCTG	55
